# Native T1 is predictive of abnormal myocardial strain in hypertrophic cardiomyopathy

**DOI:** 10.1186/1532-429X-17-S1-P263

**Published:** 2015-02-03

**Authors:** Patrick J Jensen, Davis Vigneault, Theodore Abraham, Roselle Abraham, David Bluemke, Linda Chu

**Affiliations:** Department of Radiology and Imaging Sciences, National Institutes of Health, Spring Lake, MI USA; The College, University of Chicago, Chicago, MI USA; Institute of Biomedical Engineering, University of Oxford, Oxford, UK; School of Medicine, Division of Cardiology, Johns Hopkins University, Baltimore, MD USA; Department of Radiology and Radiological Science, Johns Hopkins University, Baltimore, MD USA

## Background

Hypertrophic cardiomyopathy (HCM) is an autosomal dominant inherited disease, characterized by myocardial wall thickening in the absence of other etiologies of hypertrophy. Histologically, HCM is characterized by myocyte hypertrophy and disarray with collagen deposition. Native T1 time of the myocardium is increased in the presence of myocardial fibrosis. The purpose of this study was to determine if greater T1 time was associated with abnormal myocardial wall strain.

## Methods

Ninety-two patients were included in this cross-sectional study, all of whom had undergone CMR examination including steady state free precession (SSFP) cine and modified look-locker inversion recovery (MOLLI) imaging. Native T1 time was measured from short axis (SA) MOLLI images in the midslice of the left ventricle (LV). Strain, native T1 time, and wall thickness were measured at matched slice locations. Strain was measured using feature tracking of SSFP cine images (Multimodality Feature Tracking (MTT) algorithm v6.1.4826, Toshiba Medical Systems Corporation). Univariate comparisons were performed using Pearson's correlation and Student's t-test as appropriate, with p<0.05 considered significant. In multivariate analysis, a generalized linear model was used to control for age, gender, LV mass index, maximum LV wall thickness, and LV ejection fraction. LV mass index was defined as LV mass divided by BSA. All statistical analyses were performed using R (v3.1.0, R Core Team) and RStudio (v0.97.551, RStudio).

## Results

Of our cohort, 30% of the patients were women, 13% were African American, and 26% were non-Caucasian. The average age was 53 years. In multivariate analysis (Table), gender, ejection fraction, and native T1 time were predictive of myocardial strain (p<0.05 for all), but myocardial wall thickness was not predictive of native T1 time. Greater native T1 time was associated with lower radial (p<0.01) and circumferential (p < 0.01) strain magnitude. Per 10 milliseconds increase in native T1 time, the magnitudes of radial and mid circumferential strain decreased by 1.9% and 0.3%, respectively.

**Table 1:** Multivariable analysis, relationship of Native T1 and covariates to myocardial strain by CMR

	Radial Strain (%)		Mid Circumferential Strain (%)	
**Covariate**	**ß-value**	**P-value**	**ß-value**	**P-value**
Native T1 (ms)	**-0.19**	**<0.01**	**0.03**	**<0.01**
LV Mass Index (g/m^2)	0.07	0.67	0.02	0.41
Gender (ref=male)	-10.9	0.075	**2.2**	**0.021**
Maximum LV Wall Thickness (mm)	-0.99	0.35	0.15	0.36
Age (yrs)	0.01	0.93	-0.02	0.38
Ejection Fraction (%)	**1.31**	**<0.001**	**-0.25**	**<0.0001**

## Conclusions

In patients with hypertrophic cardiomyopathy, greater native T1 time was a more powerful predictor of decreased myocardial function than myocardial wall thickness. This suggests that the abnormality of myocardial strain in HCM may be related to the presence of diffuse myocardial fibrosis.

## Funding

The investigators would like to acknowledge funding support from the NIH Intramural Research Program.

**Figure 1:**
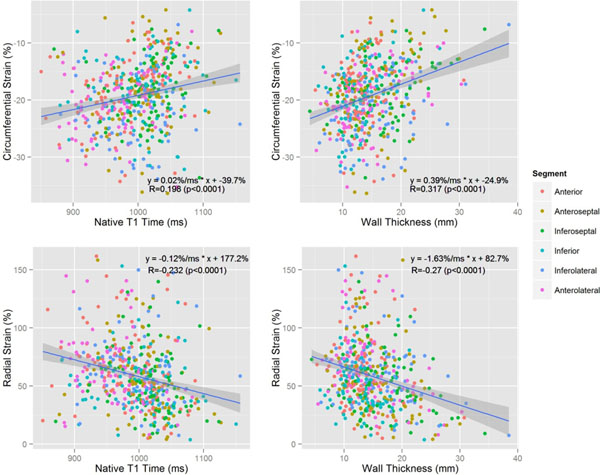
Segmental scatter plots comparing circumferential strain (top) and radial strain (bottom) to native T1 time (left) and wall thickness (right). Least squares regression nine and 95% confidence interval are indicated. The equation of the regression line, as well as Pearson's r coefficient, are indicated.

